# Switchable-Hydrophilicity Triethylamine: Formation and Synergistic Effects of Asphaltenes in Stabilizing Emulsions Droplets

**DOI:** 10.3390/ma11122431

**Published:** 2018-11-30

**Authors:** Xingang Li, Jinjian Hou, Hong Sui, Lingyu Sun, Lin Xu

**Affiliations:** 1 School of Chemical Engineering and Technology, Tianjin University, Tianjin 300072, China; lxg@tju.edu.cn (X.L.); sunnixo@163.com (L.S.); 15822806749@163.com (L.X.); 2 National Engineering Research Centre for Distillation Technology, Tianjin 300072, China; 3 Collaborative Innovation Center of Chemical Science and Engineering, Tianjin 300072, China; 4 China Academy of Safety Science and Technology, Beijing 100012, China

**Keywords:** switchable-hydrophilicity triethylamine (SHT), synergistic effect, emulsions stabilization, asphaltene

## Abstract

In this study, SHT (switchable-hydrophilicity triethylamine, [Et_3_NH]·[HCO_3_]) has been synthesized and instrumentally characterized by Fourier transform–infrared spectroscopy (FTIR) and ^13^C nuclear magnetic resonance (NMR). The operational synthesis conditions of SHT were optimized and determined at 25 °C, Et_3_N/H_2_O volume ratio of 1:2 and CO_2_ injection rate at 300 mL/min. When it was used to extract heavy oil from unconventional oil ore, it was found that it could break maltenes-in-water emulsions. When asphaltenes were present in the oil phase, it was observed that SHT could cooperate with asphaltenes. These results indicated that SHT works with asphaltenes, leading to synergistic effects in stabilizing oil–water (o/w) emulsions.

## 1. Introduction

Switchable hydrophilicity solvents (SHSs) are organic solvents with switchable miscibility between hydrophilicity and hydrophobicity [1,2]. Due to this property, SHS can be removed from the product and recycled without requiring distillation, potentially saving energy. Durelle et al. proposed a mathematical model to describe the behavior of CO_2_-triggered switchable-hydrophilicity solvents in terms of their basicity and hydrophilicity [3]. The mathematical model facilitates the optimization of the synthesis of switchable-hydrophilicity solvent by adjusting pressure and the solvent/water volume ratio.

The application of these switchable hydrophilicity chemicals as process aids in oil extraction has been widely reported [4,5,6,7,8,9,10,11,12]. For instance, SHS could efficiently extract and recovery diesel from oil-based drill cuttings [8]. SHS could extract oil from Jatropha curcas L. oil seeds to produce biodiesel, and the extraction ratio is higher than hexane [10]. Some researchers found that SHS could extract phenols from lignin bio-oil, and 91% SHS could be recovered [12]. Besides, SHSs have been used in oil–sand separation as solvents or interfacial active materials [5,6,7,13]. During this oil–solid separation, the SHSs play important roles in modifying the oil–water (o/w) interface and oil–solid interface, resulting in the formation of oil–water emulsions [5]. The formation of emulsions during oil extraction makes the separation much more difficult [14,15]. However, little information has been reported on the exact mechanisms of SHS in stabilizing oil–water emulsions, especially its role together with natural interfacial active materials, such as asphaltenes [16,17,18,19,20,21,22].

In this paper, SHT will be formed and optimized. Its role in the stabilization of maltenes-in-water emulsions will be studied. Accordingly, the objectives of this study are to (i) find the optimal conditions for the formation of SHT; (ii) to understand the synergistic effect of SHT in stabilizing oil-water emulsions together with asphaltenes.

## 2. Experimental

### 2.1. Materials

Chemicals reagents, including toluene, n-heptane, Et_3_N (triethylamine), hydrochloric acid, sodium hydroxide, and anhydrous sodium sulfate, were analytical grade purchased from Tianjin Jiangtian Technology Co. Ltd. (Tianjin, China). The Canadian oil sands samples were obtained from Athabasca, Canada. The composition of Athabasca oil sands was analyzed using the standard method to give 10.72 wt.% bitumen, 1.79 wt.% water, and 87.49 wt.% solids. The N_2_ and CO_2_ (of 99.9% purity) were purchased from Tianjin Liufang Technology Co. Ltd., China.

### 2.2. Optimization of Switchable-Hydrophilicity Triethylamine (SHT) Formation

Ten milliliters Et_3_N and 10 mL deionized water were added into to a 100 mL three-neck round-bottom flask at 20 °C in a water bath. Then, a thermometer, 5 mm-diameter gas duct and exhaust pipe were placed in the flask. After the gas duct was inserted below the surface of the water, the CO_2_ was injected into the liquid at a rate of 500 mL/min at atmospheric pressure. With the injection of CO_2_ into the flask, the pH of the water phase decreased accordingly. The pH of the solution was measured using a pH meter in real time. Initially, there were two phases due to the immiscibility of the tertiary amines in water. Continued bubbling of CO_2_ caused the phase interface to disappear, finally producing a homogeneous solution. The SHT concentration was highly dependent on the pH of the solution at a given temperature. When the pH remained stable, CO_2_ injection was stopped. This meant that the homogenous solution was formed when the pH value change was within ±0.02. Then, the SHT solution was poured into a screw-neck glass bottle, and the anhydrous sodium sulfate was put into the SHT solution until the particles floated, which indicated that the water had been removed. The process was repeated by altering the temperature, triethylamine/water volume ratio and CO_2_ injection rate according to a single factor experiment allowing the process parameters to be optimized.

### 2.3. Characterization

Fourier transform–infrared (FT–IR) spectra of SHT and Et_3_N were measured using a FT–IR spectrometer (Bruker, Karlsruhe, Germany). For nuclear magnetic resonance (NMR) sample preparation, 200 μL Et_3_N was dissolved in 800 μL of deuterated chloroform (CDCl_3_ 99.8%-d) containing tetramethylsilane (TMS 0.3% v/v), and 200 μL SHT was dissolved in 800 μL D_2_O. Subsequently, 600 μL of the mixture was transferred into a standard 5 mm NMR tube for direct measurement. The reagent was purchased from Aladdin (Shanghai, China). All one-dimensional ^13^C-NMR spectra were recorded at 500 MHz and 298 K on a Bruker AV 600 spectrometer (Bruker Corporation, Faellanden, Switzerland) equipped with a cryoprobe and a z-gradient.

### 2.4. Emulsion Stability Experiment

**Figure 1:**
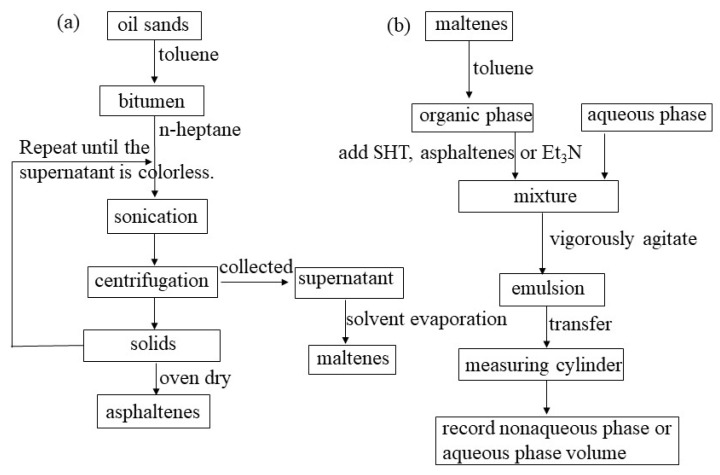
Schematic of the emulsion stability experiment procedures. (**a**) Preparation of asphaltenes and maltenes; (**b**) preparation of emulsions and the demulsification process.

The experimental procedures of the preparation of asphaltenes, maltenes, emulsions and demulsification are shown in Figure 1. The bitumen was extracted from 150 g oil sands through the toluene solvent extraction process. After extraction, the bitumen (2 g) was added to a conical flask, and 90 mL n-heptane was added. The bitumen was sonicated at 50 °C for 30 min. After sonication, the mixture was centrifuged at 8500 rpm for 15 min. After the centrifugation, the supernatant was transferred into a 500 mL conical flask. The insoluble solids were deposited back into the conical flask. The centrifugation and precipitation processes were repeated with additional 100 milliliters n-heptane until the supernatant was colorless. The n-heptane was removed by a rotary evaporator, leaving the maltenes. Then, the insoluble solids were dried in a vacuum oven at 80 °C, and the solids were the asphaltene samples. The maltenes were transferred into a flask to mix with the toluene, obtaining a mixture with maltenes at a concentration of 1.2%. The organic phase was adjusted by adding SHT, asphaltenes or Et_3_N. Twenty milliliters deionized water and 30 mL organic phase were mixed and transferred into a 100 mL round-bottom flask, and stirred at 900 rpm for 220 min. Then, the mixture was quickly transferred into a measuring cylinder, and the precipitated nonaqueous phase volume or aqueous phase volume were recorded.

### 2.5. Interfacial Tension (IFT) Measurements

The interfacial tension (IFT) between the non-aqueous phase and deionized water was measured by the pendant drop shape method (SL200B, Kino, Winslow, AZ, USA). The interfacial tension between the oil phase and water phase was measured based on the shape of a pendant drop using the asymmetric drop shape analysis technique, the similar experiment procedures could be found in the literature [6,23,24]. The temperature was 25 °C. The organic phase was injected into the water phase using a U-shape bent needle fixed on a micro-syringe. The experimental instrument could fit a theoretical Young–Laplace curve according to the drop shape. The drop got bigger when the organic phase injected by manual. The injection stopped until the drop shape was the biggest, and the drop would fall off if we continued increasing liquid when the drop shape was the biggest. Until the fitted curve was stable and unchanged, the fitted value was the IFT. The IFT of toluene/water is 36.1 mN/m, which indicated that the IFT measurement is accurate. Each measurement was repeated at least three times until the results were constant.

## 3. Results and Discussion

**Figure 2:**
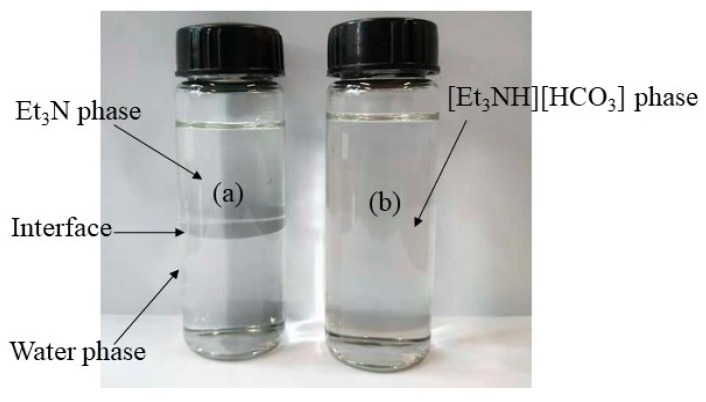
(**a**) The Et_3_N oil phase and water phase coexist in the trimethylamine-water system with an interface between them; (**b**) one homogenous phase appears in the SHT solution after CO_2_ addition.

When Et_3_N is exposed to CO_2_, it is hydrophilic and even has complete miscibility in water. The CO_2_ injection is stopped until the interface between the two immiscible phases disappears (Figure 2b), which means that the reaction reached the equilibrium state and the SHT solution formed. The equation of SHT formation is [1,24]:

(1) $${{(\mathrm{CH}}_{3}{\mathrm{CH}}_{2})}_{3}{N+H}_{2}{O+\mathrm{CO}}_{2}\overset{}{⇄}\left[{{(\mathrm{CH}}_{3}{\mathrm{CH}}_{2})}_{3}\mathrm{NH}\right]\left[{\mathrm{HCO}}_{3}\right]$$

### 3.1. Characterizations of SHT

#### 3.1.1. Fourier Transform–Infrared (FT–IR) Analysis

**Figure 3:**
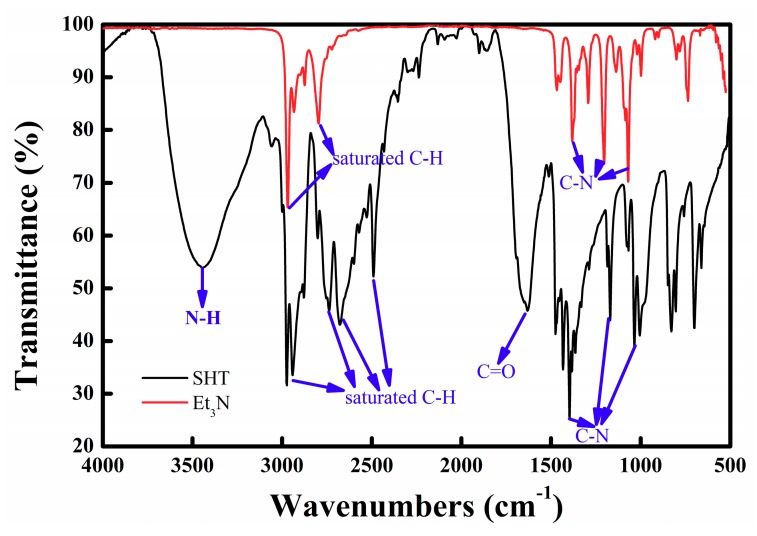
The Fourier transform–infrared (FT–IR) spectra of Et_3_N and SHT.

To further detect whether SHT has been synthesized successfully, Et_3_N and SHT were characterized by FT–IR, as shown in Figure 3. Obviously, the characteristic bands for Et_3_N were mainly located at 3000–2800 cm^−1^ (the stretching vibration of saturated C–H bonds) and 1385–1060 cm^−1^ (the stretching vibration of C–N bonds). The characteristic bands for SHT were mainly located at 3500–3400 cm^−1^, 3000–2800 cm^−1^, 1700–1600 cm^−1^ and 1500–1000 cm^−1^. The strong and broad band at 3445 cm^−1^ was associated with the –N–H stretching vibration in [(CH_3_CH_2_)_3_NH]^+^. The bands at about 2974 cm^−1^ and 2941 cm^−1^ represented the C–H asymmetrical stretching vibration of –CH_3_, –CH_2_–, respectively. In addition, the band at 1631 cm^−1^ was due to asymmetric C=O stretching vibration, which was from the [HCO_3_]^−^. The weak bands at about 1475 cm^−1^ and 1397 cm^−1^ represented the C–H scissoring vibration of –CH_2_–, and asymmetrical deformation vibration of –CH_3_, respectively. The other bands in the 1500–1000cm^−1^ range were due to the –C–N stretching vibration.

#### 3.1.2. ^13^C Nuclear Magnetic Resonance (NMR) Analysis

**Figure 4:**
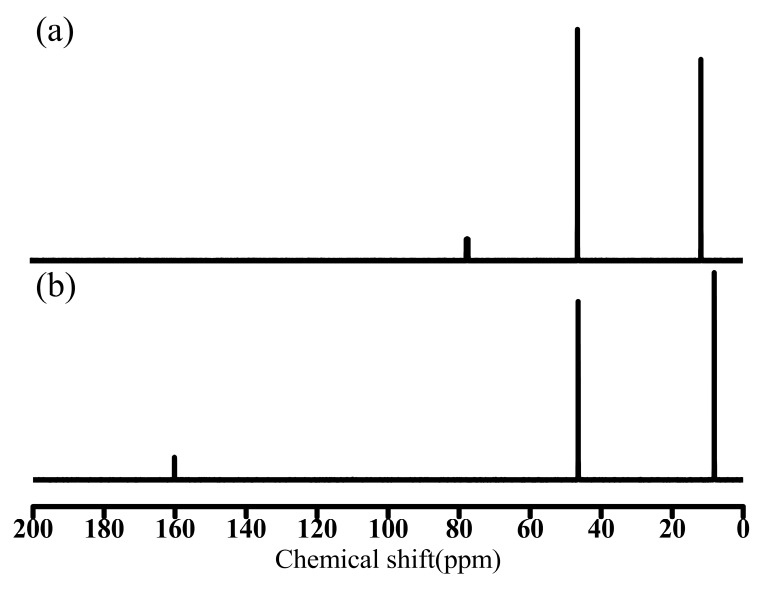
The ^13^C nuclear magnetic resonance (NMR) spectra of (**a**) Et_3_N in CDCl_3_, (**b**) SHT in D_2_O.

Et_3_N and SHT were characterized by ^13^C-NMR spectra, as shown in Figure 4. The chemical shifts of Et_3_N at 11.78 and 46.40 ppm were assigned to the –CH_3_ and –CH_2_– groups, the solvent peak is found at 77.33 ppm (Figure 4a). The chemical shifts of SHT at 8.13 and 46.47 ppm were assigned, respectively, to the –CH_3_ and –CH_2_– groups of [(CH_3_CH_2_)_3_NH]^+^ while that at 160.15 ppm was attributed to the carbon atom of [HCO_3_]^−^ (Figure 4b). These indicated that SHT has formed successfully.

### 3.2. Optimization of the Formation Process

Equation (2) describes the percentage (P) of protonated tertiary amines [3]. The protonation is dependent on solution pH and the acid dissociation constant (K*_a_*_H_) of Et_3_N. It is only related to the [H]^+^ concentration at the specific temperature for Et_3_N; therefore, pH is the indicator of reaction equilibrium.

(2) $$P=\frac{{\left[H\right]}^{+}}{{\left[H\right]}^{+}{+K}_{aH}}\times 100\%$$

**Figure 5:**
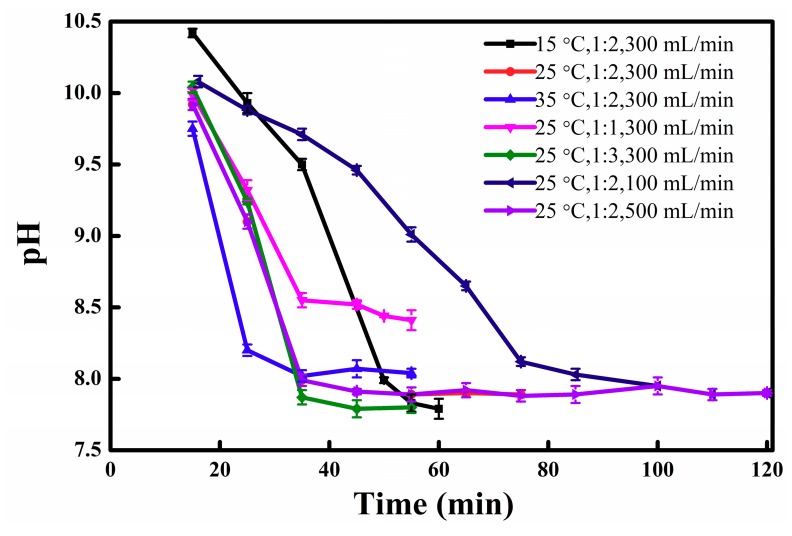
The variation of solution pH as a function of reaction time at different temperatures, Et_3_N/H_2_O volume ratios, and CO_2_ injecting rates.

The temperature and the concentration of reactants influence the chemical equilibrium and reaction rate. The temperature, amine/water volume ratio and CO_2_ injection rate are optimized (Figure 5). When the temperature increases from 15 °C to 35 °C, the reaction rate increases sharply, then pH decreasing slope is increasing with the temperature increase, so when the reaction did not reach equilibrium, the pH (35 °C) is lower than the pH (15 °C) when the reaction time is less than 45 min. Because the reaction is exothermic, when the temperature increases the conversion decreases, therefore the equilibrium pH of the solution increases. When the reaction reaches equilibrium, the pH was shown that black line (15 °C) < red line (25 °C) < blue lines (35 °C). The optimal temperature is determined to be 25 °C. With increasing Et_3_N/water volume ratio, the reaction rate sharply increases, allowing the ultimate pH to be decreased. This suggests that the conversion increases. However, when the Et_3_N/water volume ratio is less than 1:2, the pH changes little. Therefore, the optimal Et_3_N/water volume ratio is 1:2. The reaction rate also increases with the increase of the CO_2_ injection rate, but CO_2_ conversion decreases. When 10 mL Et_3_N is used and the reaction equilibrium is reached, 1.6 L CO_2_ is in theory needed. The equilibrium time for 100 mL/min was 110 min, but for 300 mL/min it was 55 min. The total CO_2_ supply at equilibrium time for 100 mL/min and 300 mL/min was 11 L and 16.5 L, respectively; the CO_2_ availability was 15% and 10%, respectively. The reaction rate accelerates with the higher CO_2_ injecting rate, and then the equilibrium time decreases. The CO_2_ availability decreases when the CO_2_ injection rate increases. When the CO_2_ is 500 mL/min, the line (was not shown in Figure 5) was similar to the red line (300 mL/min), so increasing CO_2_ injection rate to 500 mL/min was meaningless. The optimal conditions are as follows: temperature of 25 °C; tertiary amine to water volume ratio of 1:2; CO_2_ gas injecting rate of 300 mL/min.

### 3.3. The Role of SHT in Emulsion Stabilization

**Figure 6:**
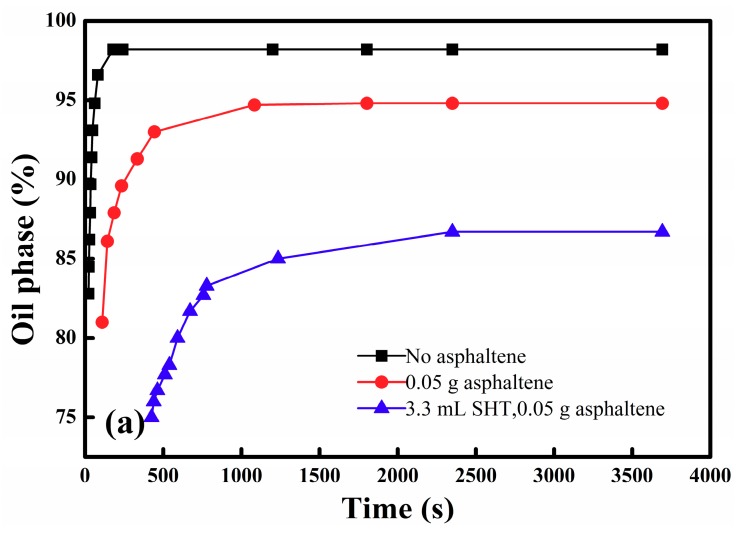

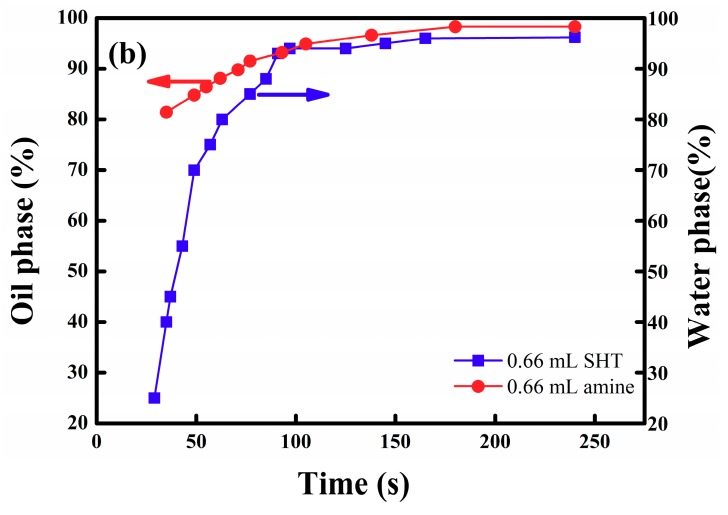
The effect of (**a**) asphaltene (1.7 g/L) and SHT content SHT (11%); (**b**) SHT (2.2%) (without asphaltenes) and Et3N (2.2 %) (without asphaltenes) on the stability of maltenes-in-water emulsions.

**Figure 7:**
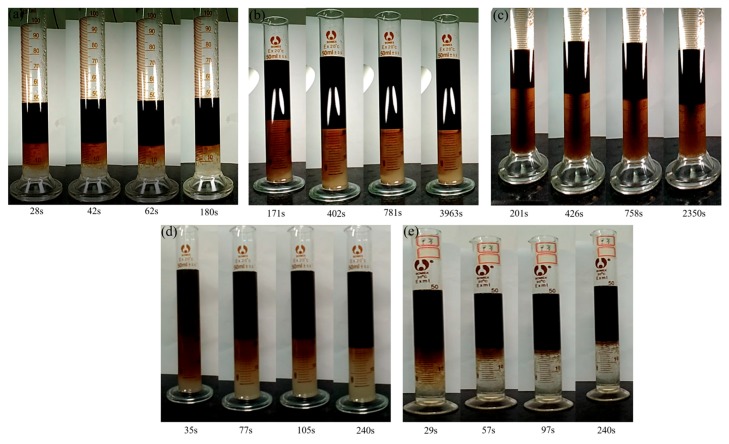
Visual appearance of emulsions at room temperature (**a**) without asphaltenes, SHT, and Et_3_N; (**b**) asphaltenes (1.7 g/L); (**c**) SHT (11%) and asphaltenes (1.7 g/L); (**d**) Et3N (2.2%); (**e**) SHT (2.2%).

The role of asphaltenes in stabilizing maltenes-in-water emulsions can also be ascertained by comparing the emulsions with different asphaltene contents, as shown in Figure 6a and Figure 7a–c. It can be seen that the emulsions made by the organic phase containing asphaltenes are much more stable. The rate of demulsification becomes slower and the amount of emulsions increases when the asphaltene content in the oil phase increases. Asphaltenes formed a rigid skin at the oil–water interface, allowing the stability of the emulsions to be improved [25,26].

The role of Et_3_N or SHT in stabilizing maltenes-in-water emulsions is shown in Figure 6b and Figure 7d,e. Since the demulsification of maltene–water emulsions by SHT is too fast to observe, the water phase is used to quantify the volume of emulsions. Comparing Figure 7d,e, SHT could accelerate demulsification but Et_3_N could stabilize emulsions. After SHT dissolves into the aqueous phase, the anion and cation compress the double electric layer. As a result, the zeta potential decreases, leading to a reduction in the interaction force. It is easy for these oil droplets to collide and coalesce under van der Waals force and, therefore, the emulsions are broken. Comparing Figure 7a,e, SHT shows the demulsification effect, and the theory is similar to electrolyte demulsification. Although SHT could individually accelerate the demulsification process, the synergistic effect of SHT and asphaltenes on stabilizing maltenes-in-water emulsions is obvious (as shown in Figure 6a).

### 3.4. IFT of the Oil and Water

**Figure 8:**
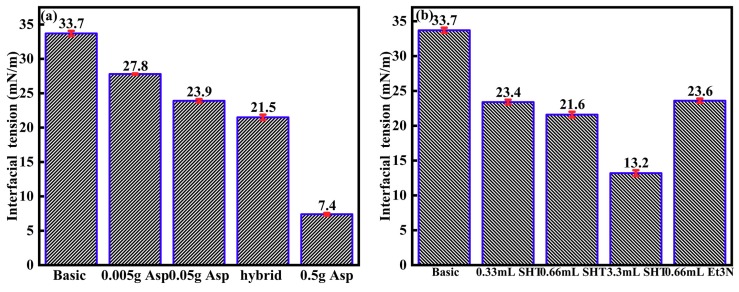
The oil–water interfacial tension as a function of asphaltenes content, SHT content, and Et_3_N content at ambient conditions (“hybrid” means 0.33 mL SHT and 0.05 g asphaltenes).

As shown in Figure 8, the interfacial tension decreases from 33.7 mN/m to 7.4 mN/m, when the asphaltene content increases from 0 to 17 g/L. Asphaltenes can adsorb at the oil-water interface; they form a protective layer; the oil-water interfacial tension decreases, and the layer could stabilize emulsions [25,27]. The interfacial tension decreases from 33.7 mN/m to 13.2 mN/m, when the SHT content increases from 0 to 11%. SHT works as a surfactant because it can decrease the oil–water interfacial tension, and this phenomenon can be found in the literature [6]. SHT enhances the adsorption of asphaltenes at the o/w interface, because the interfacial tension decreases from 23.9 mN/m to 21.6 mN/m when SHT is added to the asphaltene solution. Et_3_N could decrease the o/w interfacial tension from 33.7 mN/m to 23.6 mN/m. SHT easily resolves Et_3_N under N_2_, and both SHT and Et_3_N can decrease the oil–water interfacial tension. The combination of asphaltenes and SHT is observed to be helpful for the reduction of the oil–water interfacial tension.

### 3.5. The Mechanism of SHT and Asphaltenes’ Synergistic Effect on Stabilizing the Emulsions

**Figure 9:**
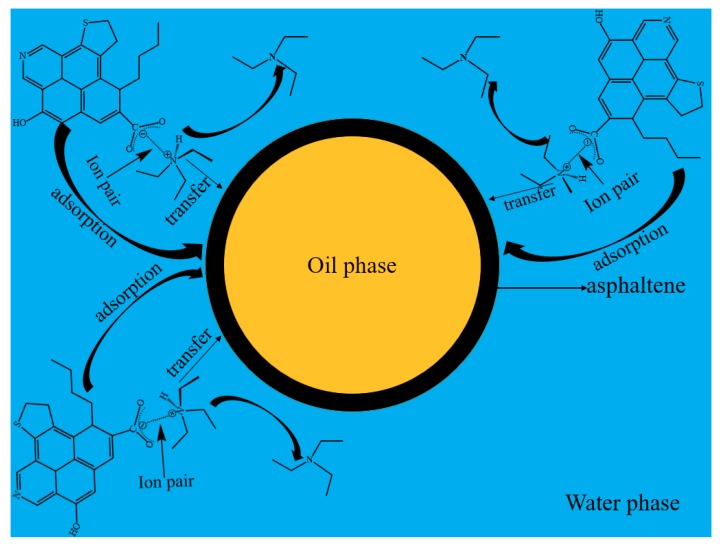
The mechanism of the synergistic effect between SHT and asphaltenes in stabilizing the emulsions.

The synergistic effect of SHT and asphaltenes on stabilizing maltenes-in-water emulsions is shown in Figure 9. SHT can help asphaltenes to adsorb at the o/w interface by forming ion pairs. It is easy to transfer the oil–water interface and this helps asphaltenes adsorb at the o/w interface. Asphaltenes that accumulate at the oil-water interface could hinder demulsification. Asphaltenes could adsorb at the oil–water interface by themselves, and the presence of SHT will facilitate the stability of the oil–water emulsions.

## 4. Conclusions

The formation conditions for SHT have been optimized by operational tests. The optimal conditions are determined at 25 °C with the triethylamine to water volume ratio of 1:2, and the CO_2_ injecting rate at 300 mL/min. It is found that SHT and Et_3_N can work as a surfactant to decrease the oil–water interfacial tension. However, only Et_3_N can stabilize the emulsions, while SHT can help to break the maltene–water emulsions. When asphaltenes are present in the oil, SHT is found to be able to work together with asphaltenes to stabilize maltenes-in-water emulsions. The improvement in the stability of emulsions by SHT and asphaltenes can be mainly attributed to the interaction between SHT and asphaltenes by forming [Et_3_NH]^+^-asphaltene ion pairs at the oil–water interface.
